# Non-Contact Heart-Rate Measurement Method Using Both Transmitted Wave Extraction and Wavelet Transform

**DOI:** 10.3390/s21082735

**Published:** 2021-04-13

**Authors:** Zheng Yang, Kazutaka Mitsui, Jianqing Wang, Takashi Saito, Shunsuke Shibata, Hiroyuki Mori, Goro Ueda

**Affiliations:** 1 Nagoya Institute of Technology, Nagoya 466-8555, Japan; y.so.446@nitech.jp (Z.Y.); k.mitsui.457@nitech.jp (K.M.); 2 Soken, Inc., Nisshin, Aichi 470-0111, Japan; takashi.saitou.j7v@soken-labs.co.jp (T.S.); shunsuke.shibata.j6b@soken-labs.co.jp (S.S.); hiroyuki.mori.j3y@soken-labs.co.jp (H.M.); 3 Denso Corporation, Kariya, Aichi 448-8661, Japan; goro.ueda.j6h@jp.denso.com

**Keywords:** heart-rate detection, non-contact, transmitted field, wavelet transform

## Abstract

Continuous monitoring of heart-rate is expected to lead to early detection of physical discomfort. In this study, we propose a non-contact heart-rate measurement method which can be used in an environment such as driver heart-rate monitoring with body movement. The method is based on the electric field strength transmitted through the human body that changes with the diastole and systole of the heart. Unlike conventional displacement detection of the skin surface, we attempted to capture changes in the internal structure of the human body by irradiating the human body with microwaves and acquiring microwaves that pass through the heart. We first estimated the electric field strength transmitted through the heart using three receiving sensors to reduce the body movement effect. Then we decomposed the estimated transmitted electric field using stationary wavelet transform to eliminate significant distortion due to body movement. As a result, we achieved an estimation accuracy of heart-rate as high as 98% in a verification experiment with normal body movement.

## 1. Introduction

In recent years, fatal accidents due to the health condition of automobile drivers have been on the increase. A sudden change in physical condition is likely to cause a serious accident, and a prompt countermeasure is required [1,2,3,4]. By grasping the health condition of the driver, it is expected that when an accident such as loss of consciousness or inability to drive occurs, the vehicle will be parked in a safe place to prevent a traffic accident. The monitoring of such biological conditions not only prevents accidents but also supports autonomous driving. The number of fatal accidents caused by health conditions is most often caused by heart disease. Monitoring the heart condition can prevent serious accidents associated with heart disease. Heart-rate is one of the indicators for grasping the condition of the heart. Knowing the driver’s heart-rate can help identify signs of heart disease such as myocardial infarction or heart failure. Continuous monitoring of the driver’s heart-rate is therefore necessary. There is a contact method in which electrodes are attached to the skin as an acquisition method such as electrocardiogram measurement [5,6]. It is, however, not suitable for long-term measurement because it causes stress. Several capacitive coupling methods have been reported for non-contact measurement of heart-rate, but they suffer from weak detection signals [7,8,9]. Another popular method is to use microwave or millimeter wave Doppler radar as a non-contact heart-rate measurement system [10,11,12]. This method focuses on heartbeat displacement detection on skin surface, and uses ultra-wideband [13,14,15,16] or millimeter wave signals to increase spatial solution [17,18]. It is not suitable, however, for use in a vehicle because the small displacement of the skin surface is very sensitive to vehicle shake or body movement. Attempts such as multi-carrier-based Doppler radar [19] or coherent sensing technique [20] have been reported to reduce the body movement effect, but their effectiveness in vehicle environments remains unclear.

In this study, we propose a method to obtain the heart-rate by using a microwave wave transmitting through the human heart even when body movement occurs. Since microwaves penetrate the human body more easily than millimeter wave and ultra-wideband signals, we can get information directly on the diastole and systole of the heart in addition to the surface of the skin. The proposed method consists of two steps. First, to easily remove the body movement effect, the field component transmitting through the heart is extracted by eliminating the field component diffracted around the body surface. Then the body movement effect is removed by decomposing the transmitted field and the body movement component using stationary wavelet transform (SWT).

This paper is organized as follows. Section 2 describes the basic principle. Section 3 describes simulation results of heart-rate estimation based on transmitted field extraction, and Section 4 describes the body movement elimination results using SWT. Section 5 experimentally verifies the effectiveness of the proposed method. Section 6 concludes this study.

## 2. Basic Consideration

**Figure 1:**
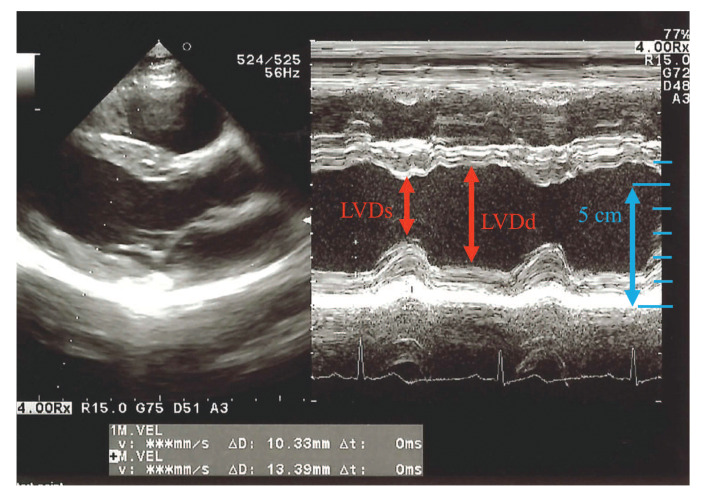
Ultrasonic image of left ventricle of the heart.

When a microwave wave is applied to the human chest, the microwave wave reaches the back by passing through the human body and diffracting around the human body. Since the internal structure of the human body changes during diastole and systole [21], it is believed that the microwave waves that reach the back change with the heartbeat. Figure 1 shows the diastole and systole of the left ventricle of the heart on the time axis by ultrasonic measurement. The left ventricle has a part where the anterior wall and posterior wall are contracted. This part is where the heart is beating and there is a displacement of 2 cm in total in the front and back. Since the human body structure around the heart changes due to this diastole and systole, it is possible to capture changes in the internal structure of human body by irradiating the human body with microwaves and acquiring microwaves that pass through the heart. To confirm this consideration, an electromagnetic field simulation with an anatomically-based human body model was performed. The numerical human body model consists of more than 50 tissue types with a resolution of 2 mm [22]. Here this model was used for diastole. On the other hand, the diameter of the heart of the human body model was reduced by 2 cm, and the part caused by the reduction was filled with air instead. This model was used for systole. The electromagnetic field simulation was performed using the finite difference time domain (FDTD) method [23] for the two models. The dielectric properties were cited from Gabriel [24].

**Figure 2:**
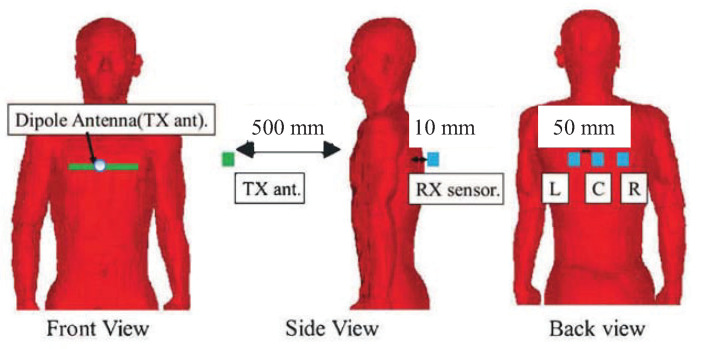
FDTD simulation environment. Tx ant: transmitting antenna.

Figure 2 shows the FDTD simulation environment. A 920 MHz sine wave was radiated form a horizontally directed half-wave dipole antenna in the front of the human body with a height of the chest and a distance of 50 cm from the body. This arrangement assumed that the antenna is set to the steering wheel position. The electric field distributions in a vertical plane with a distance of nearly 1 cm behind the human body were calculated for the diastolic model and the systolic model, respectively, and then a difference between them was obtained. This observation plane corresponded to the position of the backrest of driver’s seat. The electric field detected behind the human body is a vector quantity, which is composed of the sum of the transmitted field and the diffracted field, and the latter is dominant. It is, therefore, important which field vector to detect. There are two choices for the transmitting antenna arrangement. One is the horizontal arrangement, and the other is the vertical arrangement. Since the human body is a lossy dielectric medium, the electric field component normalized to the human body is dominant [25]. When a horizontal electric field is incident from the front of the human body, the diffracted electric field is directed in the opposite direction behind the body and cancels each other. However, when a vertical electric field is incident from the front of the human body, the diffracted electric field will have the same direction behind the body and is added. From the point of view of reducing the influence of the diffracted field because it is not affected by the diastole and systole of the heart, a horizontal antenna was used as the transmitting antenna and the sensors at the observation plane were also designed to detect the horizontal electric field components.

**Figure 3:**
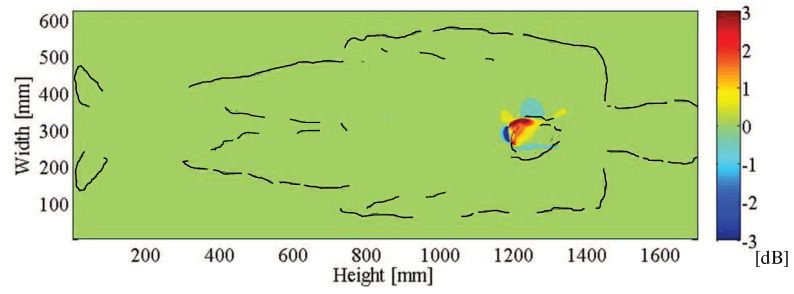
Distribution of difference of horizontal electric field strengths at a vertical plane behind the human body.

**Figure 4:**
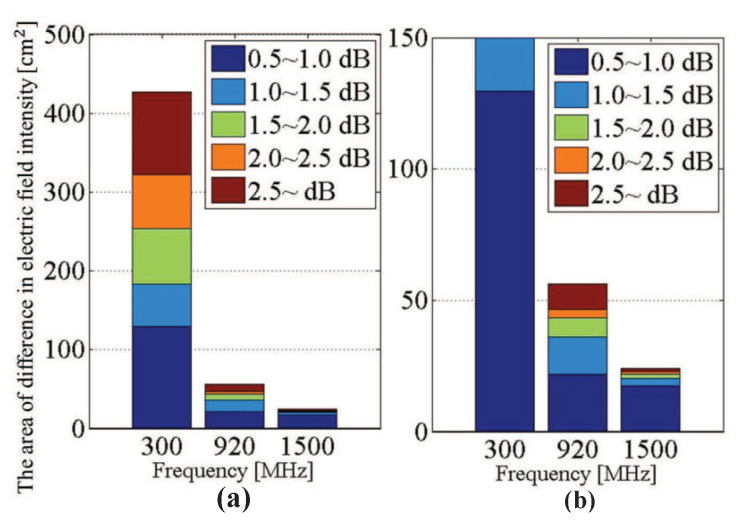
(**a**) Area of change of horizontal electric field strength behind the human body for the diastolic model and the systolic model. (**b**) An enlargement of (**a**).

Figure 3 shows the distribution of the difference of horizontal electric field strengths. From Figure 3, it is found that the electric field passing through the human body changes due to the diastole and systole of the heart, and the change can be observed at the back of the body. This result suggests that the diastole and systole of the heart can be captured by radiating microwave waves from the front of the human body, acquiring the time variation of the electric field near the heart on the back side, and processing it appropriately. In addition, the microwave was changed from 300 MHz to 920 MHz and 1500 MHz to find an appropriate frequency. The same simulation was performed, and the electric field distributions behind the back of the body for the diastolic model and the systolic model were compared. Figure 4 shows the areas in which a change of 0.5 dB or more on the horizontal electric field strength was found at each frequency. From Figure 4, it is desirable to use 920 MHz because it changes over 0.5 dB over a sufficiently large area of 50 cm${}^{2}$ and the antenna length is much smaller than 300 MHz.

## 3. Extraction of Transmitted Field

### 3.1. Formulation

**Figure 5:**
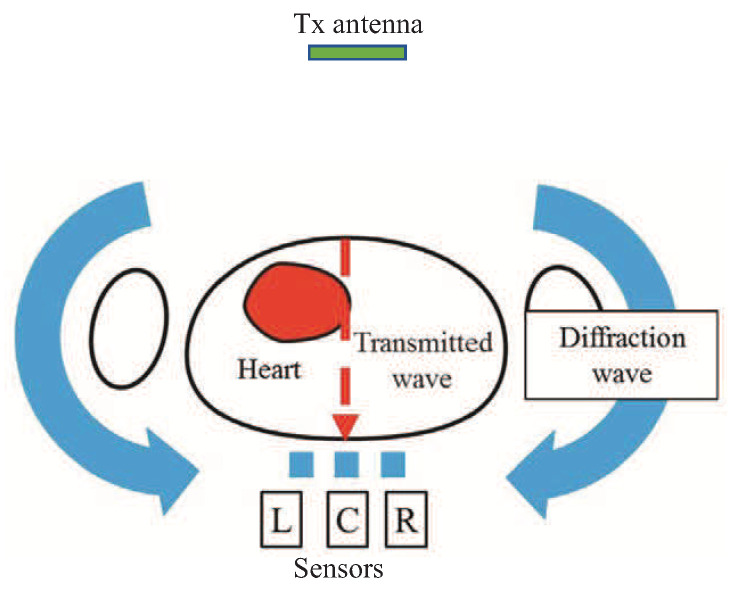
Arrangement of transmitting antenna and receiving sensors for heart-rate measurement.

Figure 5 shows the arrangement of transmitting antenna and receiving sensors for heart-rate measurement. The transmitting antenna is a 920 MHz half-wave dipole with horizontal arrangement. The three receiving sensors are located nearly 1 cm behind the body, with a 5 cm spacing between them to detect the horizontal electric field components. The three receiving sensors are named L, C, R from the left for convenience. The electric field observed at each sensor is represented by the sum of the transmitted field and the diffracted field, and the latter is not significantly affected by the diastole and systole of the heart. If the diffracted field is removed, the transmitted field can be extracted, the influence of body movement can be reduced, and the heart-rate can be estimated from the transmitted field. As an approximation, the received electric field at the three sensors can be considered to be a sum of the transmitted and diffracted fields, i.e.,

(1) $$\dot{E_{L}}=E_{L_{T}}e^{j\theta_{L_{T}}}+E_{L_{D}}e^{j\theta_{L_{D}}}$$

(2) $$\dot{E_{C}}=E_{C_{T}}e^{j\theta_{C_{T}}}+E_{C_{D}}e^{j\theta_{C_{D}}}$$

(3) $$\dot{E_{R}}=E_{R_{T}}e^{j\theta_{R_{T}}}+E_{R_{D}}e^{j\theta_{R_{D}}}$$

Equations (1)–(3) are equations representing the received electric fields at sensors L, C, and R, respectively. The symbols $\dot{E_{L}}$, $\dot{E_{C}}$ and $\dot{E_{R}}$ mean complex electric fields. The subscripts T and D mean the transmission and diffraction, respectively, and show the transmitted electric field and the diffracted electric field at each sensor. For estimating the diffracted field component at Sensor C, it is assumed that the transmitted field component in the received electric field at Sensor L or R is much smaller than the diffracted field, and the transmitted field component can be ignored. In reality, the transmitted field component at sensors L and R is difficult to ignore. However, at 920 MHz, the relative permittivity of human tissue such as muscle is 55.9 and the conductivity is 0.97 S/m, which are much larger than free space. Therefore, the electric field transmitted through the human body is greatly attenuated, and the diffracted field component on the body surface is much larger than the transmitted field component. This fact makes the above assumption roughly true. That is to say, $E_{L_{T}}e^{j\theta_{L_{T}}}$ in (1) and $E_{R_{T}}e^{j\theta_{R_{T}}}$ in (3) are ignored first. Then the diffracted field at sensor C can be represented as the sum of the electric field at sensor L whose magnitude attenuates and phase delays along the left side of the body, and the electric field at sensor R whose magnitude attenuates and the phase delays along the right side of the body. Since the human body is approximately symmetrical on the left and right sides of the body, the same attenuation factor *A* and phase delay *B* can be assumed. Then we have

(4) $$E_{C_{T}}e^{j\theta_{C_{T}}}=\dot{E_{C}}-AE_{L}e^{jB}-AE_{R}e^{jB}$$

If the attenuation factor *A* and the phase delay *B* are known, the transmitted field component at Sensor C can be extracted.

### 3.2. Parameter Estimation

First, we describe a method for quantitatively determining the attenuation factor *A* and the phase delay *B* in Equation (4). As described before, the diffracted electric field component is dominant in the electric field received by the sensors. Although the use of horizontal antenna reduces the diffracted field component at Sensor C, the human body is not perfectly symmetrical and cannot be completely canceled. In other words, the diffracted electric field component is still dominant even at Sensor C. It is, therefore, reasonable to approximate the electric field strength of the estimated diffracted field to have the same level as the electric field strength detected at Sensor C. Therefore, $|{\dot{E}}_{C}\left|/\right|\left({\dot{E}}_{L}+{\dot{E}}_{R}\right)|$ is calculated at each detection time, and the average over a time period (e.g., three seconds, because a normal heart-rate is about once per second, and three seconds maybe a reasonable choice to obtain an average) is used as the attenuation factor *A*. If *N* is the number of detected electric field data within three seconds, the attenuation factor *A* is given by

(5) $$A=\frac{1}{N}\sum_{n=1}^{N}\frac{|{\dot{E}}_{C}\left(n\right)|}{|{\dot{E}}_{L}\left(n\right)+{\dot{E}}_{R}\left(n\right)|}$$

On the other hand, regarding the phase delay *B*, the spacing between the receiving sensors is 5 cm and the wavelength of the transmitted signal at 920 MHz is 32.6 cm. So, the phases of the receiving sensors L, R, and C deviate by about 0.31π (5/32.6 × 2π) radian in free space. However, since the receiving sensors are placed less than 1 cm away from the human body, the speed *v* of the microwave propagating along the body is expected to be slower than the speed of light *c*. As an approximation, *B* is assumed as 0.63π radian on the body surface which is double of the phase delay of 0.31π in free space. The validity of this assumption will be examined in the following simulations.

### 3.3. Simulation Result

**Figure 6:**
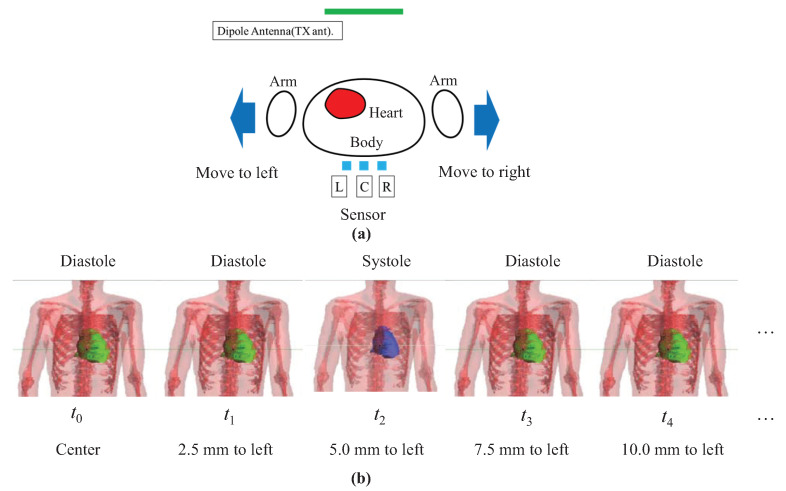
(**a**) Model for simulating the diastole and systole of the heart with body movement; (**b**) Body moving to the left.

To verify the validity of the above-described extraction method of the transmitted electric field, an electromagnetic field simulation was first performed. Figure 6 shows the model for simulating the diastole and systole of the heart with body movement. The transmitting antenna was a horizontal half-wave dipole at 920 MHz in front of the human body at a distance of 50 cm. The three receiving sensors were assumed to be behind the human body, 1 cm away from the body and have a 5 cm spacing between the sensors L and C and the sensors R and C to detect the horizontal electric field component. The human body model was assumed to move up to 30 mm to the left or right side in 2.5 mm increments. That is, the body starts at the center of Figure 6a and moves to the left side in 2.5 mm increments. When it reaches a maximum of 30 mm at the left side, it moves towards the right in 2.5 mm increments up to reaching a maximum of 30 mm at the right side. This is repeated cyclically, and the frequencies of body movement were set to 0.6, 0.8, and 1.0 Hz, respectively. This movement assumes a movement of the driver’s body due to the vibration of vehicle while driving.

**Figure 7:**
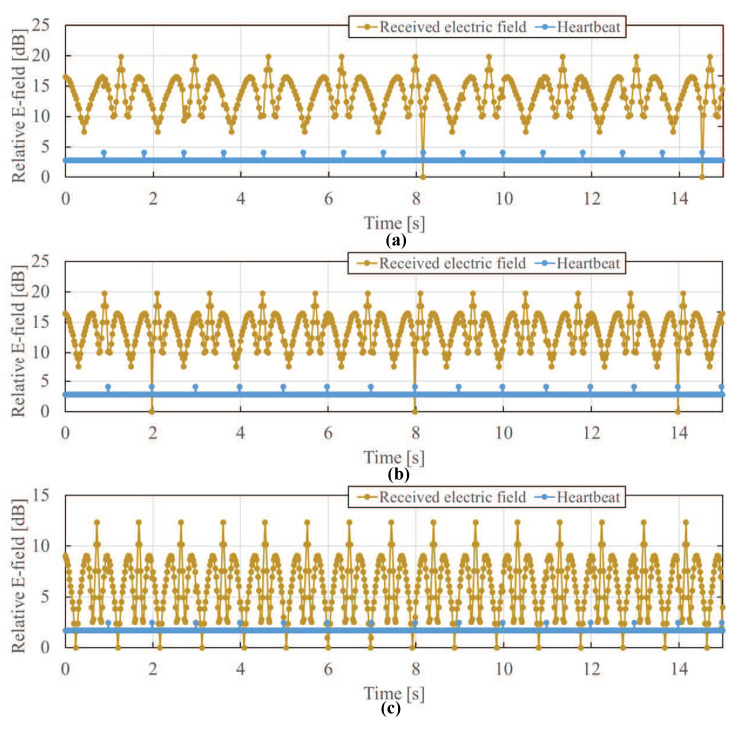
Time waveform of received electric field at Sensor C. The yellow line is the electric field detected by the Sensor C, and the blue line is the assumed heartbeat timing. The peak generated by the diastole and systole of the heart is 1 dB or more at the assumed heartbeat position. The other positive and negative peaks correspond to the positions where the body movement direction switches from left to right or from right to left. Body movement frequency: (**a**) 0.6 Hz, (**b**) 0.8 Hz, (**c**) 1.0 Hz.

The FDTD method together with the anatomical human body model was used to calculate the electric field distribution at each situation as described above. The sampling frequency at which the electric field was measured by the three sensors was 40 Hz at maximum. From Figure 2, it can be seen that the change in the electric field strength due to the diastole and systole of the heart can be captured if the receiving sensor is properly set near the back of the heart. Figure 7 shows the time waveform of the received relative electric field strength at Sensor C. The yellow line is the electric field detected by the Sensor C, and the blue line is the assumed heartbeat timing. The heartbeats were assumed by setting the diastole and systole in a fixed interval in the simulation. The peak generated by the diastole and systole of the heart is 1 dB or more at the assumed heartbeat position. As can be seen from the figure, even if the effect of the diastole and systole of the heart is observed in the detected electric field, it is still difficult to obtain the heart-rate due to the influence of body movement. It is considered that the received electric field is dominated by microwaves that are diffracted by the human body. The diffracted electric field is not affected by diastole and systole of the heart, rather than the transmitted electric field which penetrates the heart and are greatly affected by the heartbeat.

**Figure 8:**
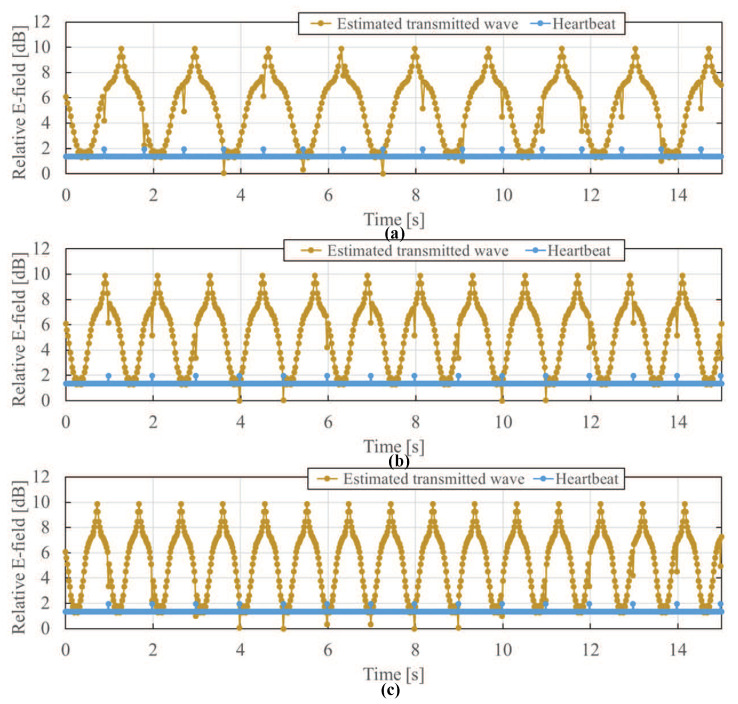
Time waveform of extracted transmitted electric field at Sensor C. The yellow line is the transmitted electric field, and the blue line is the assumed heartbeat timing. The peak generated by the diastole and systole of the heart is 1 dB or more at the assumed heartbeat position. The other positive and negative peaks correspond to the positions where the body movement direction switches from left to right or from right to left. Body movement frequency: (**a**) 0.6 Hz, (**b**) 0.8 Hz, (**c**) 1.0 Hz.

Using the above-described extraction method of transmitted field, the transmitted electric field was extracted by removing the diffracted electric field component. Figure 8 shows the extracted transmitted electric field at Sensor C. The yellow line is the transmitted electric field, and the blue line is the assumed heartbeat timing. Compared to Figure 7, the electric field distortion due to body movement is reduced in the extracted transmitted electric field, and the relative variation in strength is reduced from 13 dB to 8 dB approximately. It is also found that the time waveform of the extracted transmitted electric field has sharp positive and negative peaks only at the position where the body movement direction switches from left to right or from right to left, except for the position with respect to the heartbeat. Therefore, the extraction of the transmitted electric field is valid to measure the heart-rate by suppressing the influence of the diffracted electric field due to body movement.

From Figure 7 or Figure 8, the heart-rate was obtained from the number of peaks within one minute. A peak was defined as a position where there was a difference of more than 0.5 dB from the adjacent values. Table 1 shows the estimation results from the received electric fields at Sensor C before and after the transmitted field extraction. It can be seen that if the electric field detected at Sensor C is directly used without using the transmitted field extraction by removing the diffracted field, the peaks due to the body movements are also detected so that the heart-rate is largely over estimated. The faster the movement of the body, the higher the estimated heart-rate is. However, by extracting the transmitted electric field at Sensor C as proposed, the heart-rate has been obtained in a high accuracy. This result shows that the proposed method using three sensors to remove the diffracted field and extract the transmitted field at Sensor C is valid to measure the heart-rate, and the estimated attenuation factor *A* and phase delay *B* are also reasonable.

## 4. Improvement Using SWT

In the previous section, we described a heart-rate estimation method by extracting the transmitted electric field behind the human body and showed its usefulness by simulation. However, the waveform distortion due to body movement was not completely removed. There is a risk that the heart-rate may be erroneously detected due to sudden body movement. The time waveform of the transmitted electric field is a waveform in which the distortion due to the body movement and the change of the electric field strength due to the diastole and systole of the heart are combined. Therefore, the estimation of heart-rate would be more accurate if the distortion due to body movement and the waveform of heartbeat can be separated. This problem may be solved by using wavelet transform which is a time-frequency analysis method. The wavelet transform uses a wavelet function which satisfies a certain condition (called a basic wavelet) [26]. The frequency domain and time domain information of the waveform to be analyzed is obtained by scaling the basic wavelet while moving it on the time axis and correlating it with the analyzed waveform. The stationary wavelet transform (SWT) is a discrete wavelet transform with no down-sampling. In the usual discrete wavelet transform, the filter coefficient does not change regardless of the decomposition level. However, the SWT does up-sampling by interpolating 0 between the coefficients as the decomposition level increases [27]. This feature is useful for reducing the distortion of the transmitted electric field due to body movement.

**Figure 9:**
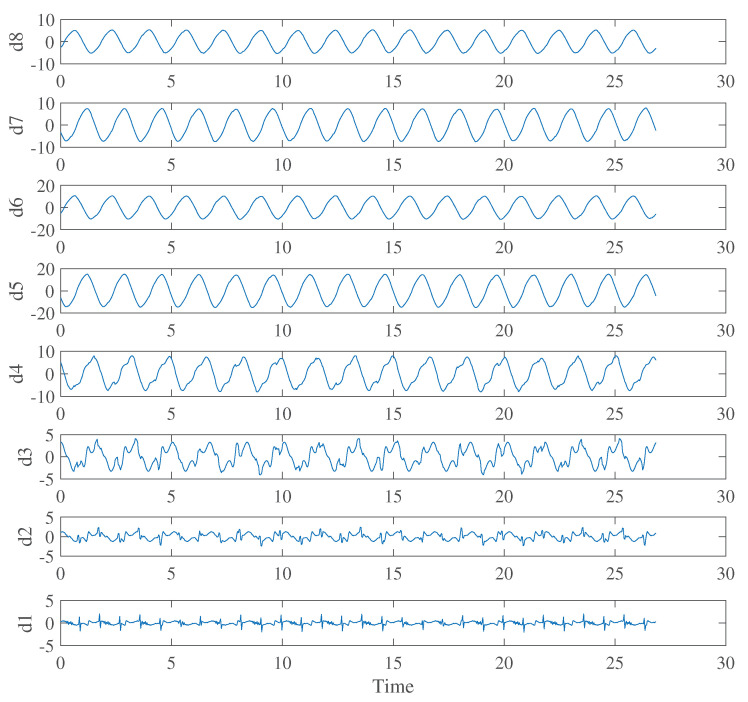
SWT of the extracted transmitted electric field at Sensor C when the body movement frequency is 0.6 Hz.

**Figure 10:**
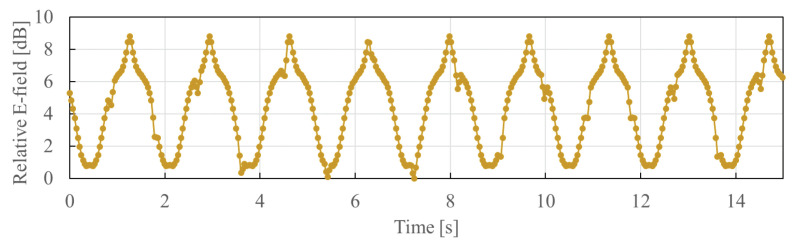
Body movement waveform separated using SWT when the body movement frequency is 0.6 Hz.

To separate the body movement component and the heartbeat component of the transmitted electric field waveform using SWT, we set the order of SWT to 8 and chose the basic wavelet as Haar wavelet. Figure 9 shows the waveforms obtained by applying SWT to the transmitted electric field in Figure 8a when the body movement frequency is 0.6 Hz. From Figure 9, it was found that the heartbeat component was most visible in $d_{1}$. Therefore, the body movement waveform was created by performing the inverse SWT from $d_{2}$ to $d_{8}$, and the heartbeat component was obtained by subtracting it from the original waveform. Figure 10 shows the separated body movement waveform at a body movement frequency of 0.6 Hz. Compared to Figure 8, it can be seen that the waveform distortion due to body movement was reproduced. It should be noted that the same simulation was also performed at 0.8 Hz and 1.0 Hz. In the FDTD simulation, it is not easy to set a random body movement because it will result in a huge computation burden. We therefore set a constant body movement frequency of 0.6 Hz, 0.8 Hz, and 1.0 Hz. However, in the experimental verification in the following section, the human subjects moved more randomly than at a constant frequency.

**Figure 11:**
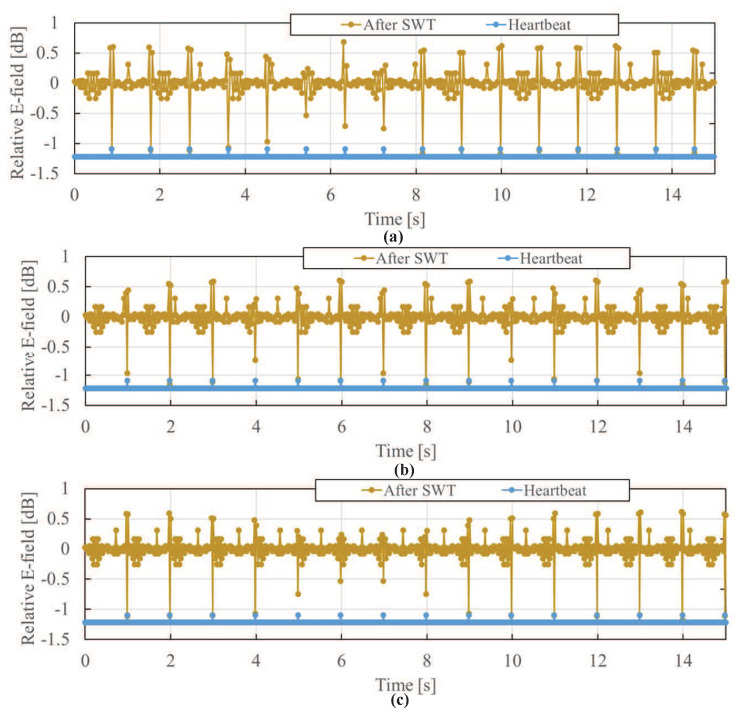
Time waveform of extracted transmitted electric field after SWT at Sensor C. Body movement frequency: (**a**) 0.6 Hz, (**b**) 0.8 Hz, (**c**) 1.0 Hz.

Figure 11 shows the time waveforms of the transmitted electric field after using SWT to reduce the distortion due to body movement. Although the peak generated due to the change of body movement direction cannot be completely removed even by using SWT, because the waveform shapes at the timing when the body movement direction changes and the timing of the heartbeats are similar, it can be seen that the waveform distortion due to body movement is greatly reduced compared to the waveform before SWT is applied, and the heartbeat is emphasized more. In Figure 11, if the relative electric field is 0.5 dB less than the values at the two adjacent timings on the left and right, the peak is considered to be due to the heartbeat. From the number of peaks due to the heartbeat within one minute, the heart-rate is obtained and its estimation error is shown also in Table 1. The estimation error of heart-rate is kept at 0%. By comparing Figure 11 with Figure 9, it can be found that SWT significantly reduces waveform distortion due to body movement and consequently results in easier heartbeat detection. To effectively estimate the heart-rate in a high accuracy, therefore, it is desirable to remove the body movement by both extracting the transmitted electric field and using the SWT.

## 5. Experimental Verification

**Figure 12:**
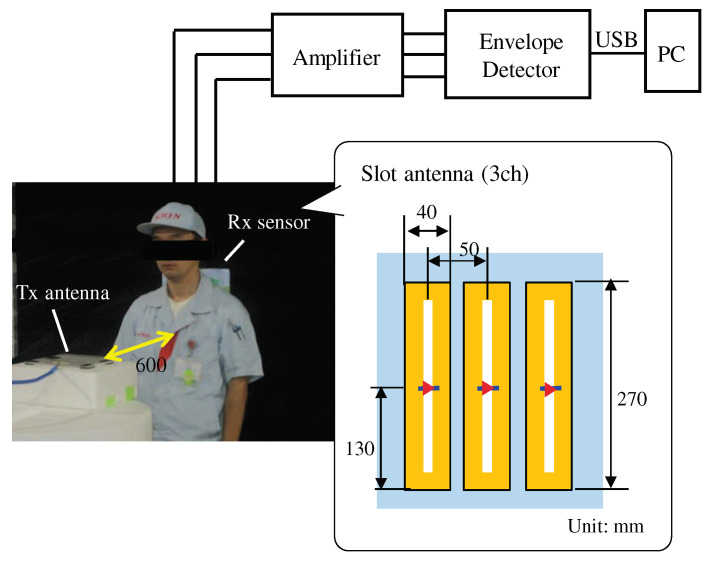
Experimental setup and receiving sensor structure.

The feasibility of the proposed method was experimentally verified. Figure 12 shows the experimental setup and the receiving sensor structure in which three slot antennas were used to detect the horizontal electric field components. The three signals at the receiving sensor were amplified, first sent to an envelope detector, and then sent to a personal computer via USB interface for applying the proposed method. Similar to the simulation, a horizontal half-wave transmitting antenna at 920 MHz was used in front of the human body and the three slot antennas were used behind the human body as sensors. This setting was based on the consideration that the transmitting antenna is set to the steering wheel and the three slot antennas are installed on the backrest of the driver’s seat. The pulse rate was also measured with a sphygmography at the same time, and was used as a reference of heartbeat. Based on our proposed method, the transmitted electric field at the sensor was first extracted using Equation (4). The attenuation factor *A* was determined using Equation (5), and the phase delay *B* was determined by the propagation distance along the body surface. In the previous simulation, since the sensors were very close to the human body at an interval not exceeding 1 cm, it was assumed that the wavelength was shortened by a factor of 2, and *B* was set to 0.63π radian. In this experiment, however, since the sensors were placed above 1 cm from the human body, the wavelength shortening had little effect and 0.31π radian (propagation at almost the speed of light) was thus chosen as the phase delay *B*. After the extraction of the transmitted electric field, SWT was applied to the extracted transmitted electric field. Since the sampling frequency of the experiment was about 20 times that of the simulation, the order of SWT increased from 8 to 16, and the body movements generated from the wavelet coefficients except for $d_{9}$ and $d_{10}$ were removed from the extracted electric field to obtain the electric field waveform mainly including the heartbeat information. The basic principle of the coefficient section is to extract the body movement components that can be represented by performing the inverse SWT from some selected *d* coefficients. The selected *d* coefficients should have less heartbeat component (few frequency components close to the heart-rate). There is no need to use different *d* coefficients for different subjects.

**Figure 13:**
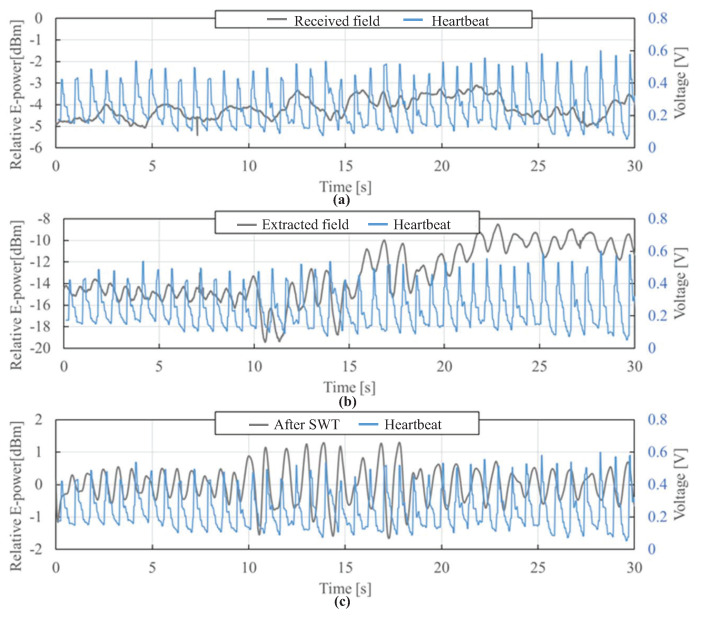
Experimental result. (**a**) time waveform of received electric field; (**b**) time waveform of extracted transmitted electric field; (**c**) time waveform of extracted transmitted electric field after SWT.

The experimental verification was performed for more than three male subjects between the ages of 30 and 50, and approved by an ethical committee in their institution (Project code KK200480). Figure 13 shows the extracted transmitted electric field at a sensor and that after SWT for the experiment data with a usual body movement while driving. From Figure 13a,b, it can be seen that the distortion of the time waveform of electric field due to body movement is reduced, and the heartbeat is emphasized. Also, as shown in Figure 13c, it can be seen that after SWT, although the peak positions in the time waveform of electric field are different, the intervals between the peaks are almost the same as the pulse wave. In addition, it is shown that the accuracy of the heart-rate obtained from the transmitted electric field after SWT is improved as shown in Table 2, and the heart-rate can be detected with an accuracy of 98% after SWT, which was calculated by the ratio of the estimated heart-rate and the reference heart-rate.

## 6. Conclusions

In this study, we have developed a non-contact heart-rate measurement method for continuously monitoring the heart-rate. The method is based on that the electric field strength transmitted through the human body changes with the diastole and systole of the heart. First, the electric field strength is recorded as a function of time using three receiving sensors behind the human body. Then the electric field transmitted through the heart is extracted by removing the diffracted electric field from the electric fields received at the three sensors for reducing the effect of body movement. Finally, the SWT is applied to the extracted transmitted electric field to further reduce the effect of body movement. As a result, the heart-rate has been estimated with an accuracy of 98% in our verification experiment for normal body movement while driving. Verification experiments in actual vehicles are a future task.

## Figures and Tables

**Table 1:** Comparison of error of heart-rate estimated from the received original electric field at Sensor C and the extracted transmitted electric field at Sensor C.

Freq.	Without Extr.	With Extr.	With Extr. & SWT
0.6 Hz	+13.8%	0%	0%
0.8 Hz	+31.6%	0%	0%
1.0 Hz	+66.7%	5.0%	0%

Freq.: Frequency of body movement;Without Extr.: using original field;With Extr.: using extracted transmitted field; With Extr. & SWT: using extracted transmitted field and SWT.

**Table 2:** Estimated heart-rate in the verification experiment.

Reference	Without Extr.	With Extr.	With Extr. & SWT
78	89	84	77

Reference: measured by a sphygmography;Without Extr.: estimated using original field;With Extr.: estimated using extracted transmitted field; With Extr. & SWT: estimated using extracted transmitted field and SWT.

## Data Availability

A part of data is available upon request.
